# Polymerized Alizarin Red–Inorganic Hybrid Nanoarchitecture (PARIHN) as a Novel Fluorogenic Label for the Immunosorbent Assay of COVID-19

**DOI:** 10.3390/bios15040256

**Published:** 2025-04-16

**Authors:** Fatema Kaladari, Mahmoud El-Maghrabey, Naoya Kishikawa, Rania El-Shaheny, Naotaka Kuroda

**Affiliations:** 1 Department of Analytical Chemistry for Pharmaceuticals, Course of Pharmaceutical Sciences, Graduate School of Biomedical Sciences, Nagasaki University, 1-14 Bunkyo-machi, Nagasaki 852-8521, Japan; 2 Department of Pharmaceutical Analytical Chemistry, Faculty of Pharmacy, Mansoura University, Mansoura 35516, Egypt; dr_m_hamed@mans.edu.eg (M.E.-M.); rania_yomna@mans.edu.eg (R.E.-S.)

**Keywords:** polymer–inorganic hybrid nanoarchitecture, non-enzymatic immunoassay, signal multiplication, fluorescence, immunochromatographic assay

## Abstract

This study seeks to develop and implement a non-enzymatic fluorescent labeling for immunoassay and immunochromatographic assay (ICAs) targeting SARS-CoV-2, to meet the extensive interest and need for effective COVID-19 diagnosis. In this manuscript, we delineate the development, synthesis, and evaluation of a novel quinone polymer zinc hybrid nanoarchitecture, referred to as polymerized alizarin red–inorganic hybrid nanoarchitecture (PARIHN), which integrates an antibody for direct use in fluorescent immunoassays, offering enhanced sensitivity, reduced costs, and improved environmental sustainability. The designed nanoarchitecture can enhance the sensitivity of the immunoassay and enable rapid results without the complexities associated with enzymes, such as their low stability and high cost. At first, a chitosan–alizarin polymer was synthesized utilizing quinone–chitosan conjugation chemistry (QCCC). Then, the chitosan–alizarin polymer was embedded with the detection antibody using zinc ion, forming PARIHN, which was proven to be a stable label with the ability to enhance the assay stability and sensitivity of the immunoassay. PARIHN can react with phenylboronic acid (PBA) or boric acid through its alizarin content to produce fluorescence signals with an LOD of 15.9 and 2.6 pm for PBA and boric acid, respectively, which is the first use of a boric acid derivative in signal generation in the immunoassay. Furthermore, PARIHN demonstrated high practicality in detecting SARS-CoV-2 nucleoprotein in fluorescence (PBA and boric acid) systems with an LOD of 0.76 and 10.85 pm, respectively. Furthermore, owing to the high brightness of our PARIHN fluorogenic reaction, our labeling approach was extended to immunochromatographic assays for SARS-CoV-2 with high sensitivity down to 9.45 pg/mL.

## 1. Introduction

The global pandemic caused by SARS-CoV-2 has highlighted the urgent need for effective and reliable diagnostic tools for emerging infectious diseases. Traditional immunoassay methods, while valuable, often face limitations in sensitivity, cost, and operational complexity [1].

Researchers are now looking for faster, more sensitive, and more accurate ways to label ELISA. One way they are doing this is by using nucleic acid template target amplification for enzymatic labels such as horse radish peroxidase (HRP) [2] to strengthen signals or by using new nanomaterial labels such as single-atom nanozymes Fe-N-C [3] as creative tags. However, nucleic acid template target amplification needs time-consuming and expensive ways to carry out DNA hybridization in order to achieve signal multiplication [2]. In addition, single-atom nanozymes are time-consuming, laborious, and costly [3]. Additionally, researchers have used versatile nanomaterials as label tags for SARS-CoV-2 immunoassay, such as Au@PtNPs nanozyme linked to either immunochromatographic sensor (NLICS) [4] or in syringe filtration immunoassay [5], aptamer-based enzyme (HRP)-linked oligonucleotide [6], and Co-Fe@hemin nanozyme [7].

All of these methods rely on the enzymatic or enzyme-mimicking labeling of detection antibodies, where enzymes or nanozymes generate reactive oxygen species (ROS) that react with specific substrates to produce colorimetric or chemiluminescent signals [8]. However, these systems [2,3,4,5,6,7] require high costs, are laborious, sensitive to micro-environmental changes, and require toxic and expensive substrates [9,10,11]. In addition, systems that depend on enzymatic labels [2,6] are highly prone to instability. Additionally, enzymatic and nanozyme reactions [2,3,4,5,6,7] can be inhibited by scavenger molecules, such as reducing agents and phenolic compounds [12,13].

Conversely, fluorescence technology has been extensively applied in immunoassay development, offering high detection sensitivity and a variety of measurable properties from fluorophores [14]. Fluorescence immunosorbent assays (FIAs) provide ultra-sensitivity and specificity for biomolecular detection, yielding faster results than traditional methods and facilitating quicker clinical decision making. The combination of sensitivity, specificity, and efficiency makes fluorescent immunoassays powerful tools in both research and clinical applications [15]. Moreover, polymer-based fluorescent labeling can detect low concentrations of antigens or antibodies due to amplified fluorescent signals, making them suitable for early disease detection.

Hybrid organic–inorganic nanomaterials containing metals and organic components have inspired biomimetic strategies that exhibit high stability and bioactivity. Similar to biomineralization, these materials can entrap proteins in a phosphate crystal framework to form protein–inorganic hybrid nanoarchitectures (PIHNs), which possess dual functions for target-specific recognition and significant signal amplification [16,17]. Recently, PIHNs have been employed as rapid and sensitive labels in ELISA, demonstrating improved stability and performance over traditional methods. For instance, Wei et al. enhanced ELISA performance using an antibody–enzyme–inorganic PIHN for the sensitive detection of pathogens like E. coli O157:H7 via TMB substrate [18]. Andrade et al. utilized a PIHN based on poly(vinyl alcohol) to detect bovine herpesvirus through colorimetric changes induced by O-phenylenediamine and hydrogen peroxide [19]. However, these methods remain dependent on enzymatic ROS generation, which has notable drawbacks that were mentioned earlier.

Our research group has been focusing on the use of quinones as a highly stable and cost-effective labeling tag for immunoassays [9]. Quinone’s redox cycle reactions generated reactive oxygen species, which were employed as a signal-generating tag for luminol/dithiothreitol (DTT) chemiluminescence [20] and INT/NaBH4 colorimetric assays [10,11]. These events allowed our lab to employ quinones instead of enzymes as immunoassay signal markers. The avidin–biotin system labeled the detection antibody with biotinylated quinone monomers and polymers [10,11]. However, all these approaches depended on ROS generation, which could be troublesome and susceptible to non-specific quenching in some matrices [21,22]. Additionally, an avidin–biotin system is needed, which increases immunoassay steps and time. Due to avidin’s non-specific binding to other sample components [23], the avidin–biotin system often has considerable background noise.

Herein, we propose an innovative method that combines the advantages of PIHNs and fluorescence-based ELISA for ultra-sensitivity. We designed and fabricated a non-enzymatic fluorogenic polymer–IHN, termed polymerized alizarin red–IHN (PARIHN), composed of zinc metal, antibodies, and a chitosan–alizarin polymer. This nanoarchitecture incorporates a detection antibody, enabling direct application in immunoassays.

A cost-effective and safe quinone polymer (chitosan–alizarin red) is utilized to design an enzyme-free polymer–IHN for immunolabeling. Alizarin is a natural hydroxyl anthraquinone derived from Rubia cordifolia, which is readily accessible, affordable, and non-toxic [24]. Alizarin is used here as a substitute for enzymes in immunoassay labeling owing to its unique fluorogenic reactions with boric acid derivatives [25,26]. On the other hand, chitosan is a biocompatible and biodegradable polycationic linear polysaccharide obtained through the partial deacetylation of chitin [27,28]. The characteristics of alizarin and chitosan prompted the synthesis of an alizarine biopolymer, employing chitosan as a backbone through quinone–chitosan conjugation chemistry (QCCC) [29], significantly enhances assay sensitivity while reducing costs and promoting environmental sustainability.

**Scheme 1:**
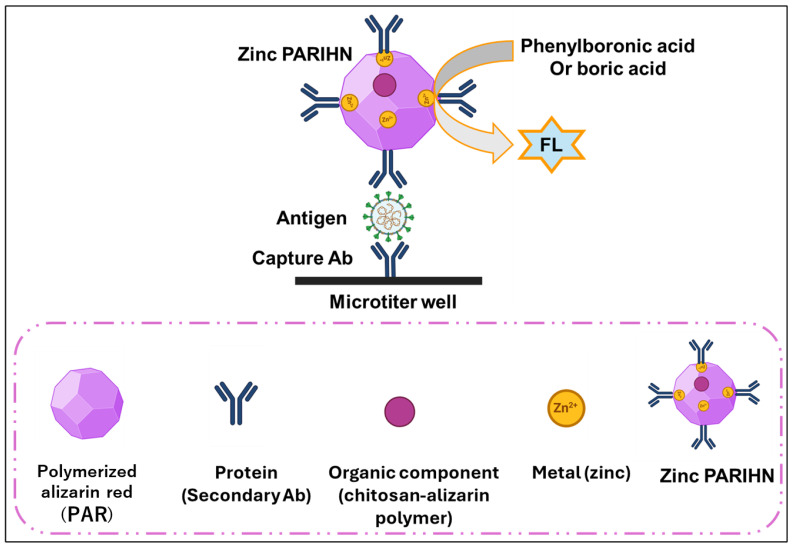
Presentation of the three main components of Zinc PARIHN, which are as follows: (1) a polymerized alizarin red; (2) a metal ion (Zn^2+^); and (3) a detecting or secondary antibody for use in a sandwich immunoassay as a signal generator for fluorescence detection.

In this study, we developed a zinc PARIHN fluorescence immunosorbent assay that generates both fluorescence and colorimetric signals through reactions with boric acid derivatives (Scheme 1). The zinc PARIHN demonstrated stability, enhanced sensitivity due to increased surface area, and maintained antibody activity, all while being synthesized via a non-toxic, straightforward method. The fluorogenic reaction of alizarin with boric acid derivatives is rapid, yielding a product with strong fluorescence at longer wavelengths. These attributes position zinc PARIHN as an excellent candidate for labeling immunosorbent assays.

Additionally, immunochromatographic assays (ICAs) have emerged as a prominent point-of-care testing approach due to their low cost, ease of operation, and rapid response. ICAs are widely used in clinical, environmental, and safety analyses, particularly during the COVID-19 pandemic [30]. This paper introduces a groundbreaking approach utilizing PARIHN as a novel fluorogenic non-enzymatic label for immunosorbent assays targeting COVID-19 and in fluorescence immunochromatographic assays as rapid tests. The performance of our PARIHN was favorably compared with conventional ELISA methods and commercially available ICA test kits, demonstrating superior sensitivity.

To our knowledge, this is the first instance of using boric acid as a reagent to generate fluorescence intensity upon reaction with zinc PIHN, achieving ultra-sensitivity, a method not previously employed in other assays. Additionally, phenylboronic acid (PBA) was utilized in the fluorescence assay, showing good sensitivity. Notably, colorimetric assays can also be performed through PBA derivatization, yielding a colored derivative measurable at 480 nm with moderate sensitivity, thus showcasing the versatility of the developed labeling approach.

## 2. Materials and Methods

### 2.1. Chemicals and Reagents

Sodium chloride, sodium bicarbonate, sodium carbonate, sulfuric acid, hydrochloric acid, sodium acetate, disodium hydrogen phosphate, acetic acid, and N-acetyl glucosamine were procured from Nacalai Tesque (Kyoto, Japan). Boric acid and Biotin-horseradish peroxidase (HRP) were supplied from Sigma-Aldrich (St. Louis, MO, USA) and Rockland Inc. (Limerick, PA, USA), respectively. Sodium alizarin sulfonate, potassium chloride, Tween 20, hydrogen peroxide, zinc acetate, and potassium dihydrogen phosphate were procured from Wako Pure Chemical Industries (Osaka, Japan). Sodium hydroxide and phenylboronic acid were procured from Merck (Darmstadt, Germany). The human SARS-CoV-2 nucleoprotein sandwich ELISA kit (Kit-LS-F74960) was acquired from Lifespan Biosciences (San Diego, CA, USA). Distilled water was produced with a Yamato Autostill WG203 (Tokyo, Japan). Phosphate-buffered saline (PBS; 100 mM, pH 7.4) was produced by combining NaCl, KCl, potassium dihydrogen phosphate, and disodium phosphate.

### 2.2. Instruments and Materials

A microplate (cell culture microplate, 96 wells, polystyrene, flat-bottomed (chimney well), black, CELLSTAR^®^, T, L.I.D., sterile, Greiner Bio-One Co., Ltd., Tokyo, Japan), a multimode microplate reader (Spectra Max M5, Molecular Devices, San Jose, CA, USA), and data processing software (Molecular Devices, Softmax^®^ Pro 5 software) were utilized in the microplate-based assays. The pH measurement was conducted using the F-71 pH meter (Horiba, Kyoto, Japan). A UV1800 UV–Vis spectrophotometer was used for spectrophotometric measurements. The preliminary fluorescence and colorimetric measurements were performed on an RF-6000 spectrofluorometer and a UV-1800 spectrophotometer from Shimadzu (Kyoto, Japan). Scanning electron microscopy (SEM) was conducted using a JSM-7500F field emission microscope (JEOL, Akishima, Tokyo). The SEM apparatus was used at a voltage of 15 kV. Aluminum sample trays (ϕ35) were used for affixing the samples for SEM measurement.

### 2.3. Synthesis of Polymerized Alizarin Red Using Deacetylated Chitosan as the Backbone

At first, deacetylation of chitosan was carried out to increase its solubility and reactivity with alizarin. The deacetylation was carried out as previously described in the literature using thermal treatment with NaOH [31,32]. Briefly, 5.0 g of low-MW chitosan (deacetylation degree 85%) was dissolved in 50 mL of 10.0 M sodium hydroxide aqueous solution and incubated at 80 °C for 8.0 h, then filtered, and the filtered precipitate was subjected to the same treatment for another two times. Finally, the precipitate was washed with water until neutral filtrate was obtained, then the precipitate was dried in an oven for one day at 105 °C. Finally, about 2.9 g chitosan with 98.0% deacetylation degree was obtained.

For the synthesis of polymerized alizarin red, 10.0 mL of chitosan (1.6 mg/mL) was reacted for 12.0 h with alizarin red (4.5 mM) in phosphate buffer with pH 6.5. Then, the pH was adjusted to 7.0, followed by centrifugation, and the polymer-containing precipitate was collected and washed with deionized water. The number of alizarin molecules in the chitosan–alizarin polymer was determined by colorimetric reaction with phenylboronic acid, as described in the following Section.

### 2.4. Determining the Number of Alizarin Number in Chitosan–Alizarin Polymer

A standard curve was plotted from the reaction of alizarin and phenylboronic acid vs. the absorbance at 480 nm. Alizarin standards 50–500 µM were added to the microplate wells, followed by the addition of 15.0 mM PBA (in PBS pH 7.4), and after 1.0 h, the absorbance was measured at 480 nm. After that, 1 mg/mL of chitosan alizarin polymer was added to the microplate well, followed by 6.0 mM PBA (in PBS, pH 7.4). The added volume of the solutions was 50 µL for each solution/sample/standard. Each experiment was repeated three times.

### 2.5. Synthesis of Zinc PARIHN with Commercially Available Secondary Ab of SARS-CoV-2 Nucleoprotein

A stock solution of 1.0 mg/mL of polymerized alizarin red in PBS (0.01 M pH 7.4) was prepared. Then, 100 µL of 1.0 mM Zn^2+^ aqueous solution was added to 1 mL stock solution and mixed, then kept for 12 h at 4 °C. Next, 60 µL of 1.0 µM SARS-CoV-2 nucleoprotein Ab in PBS (pH 7.4) was added to the mixture and kept for 24 h at 4 °C. The solution was centrifuged for 1 min and washed with deionized water three times. Then, the obtained PARIHN was diluted 100 times with a diluent solution provided by the kit and used immediately for labeling the SARS-CoV-2 nucleoprotein Ag fixed by the capture Ab.

### 2.6. Measuring the FL Intensity of PARIHN

Two reagents were used for the fluorogenic derivatization of the alizarin content in the polymer. The first reagent was boric acid, where 150 µL of PARIHN (0.75–18.7 nM) was added to the microplate wells, followed by the addition of 150 µL of boric acid (50.0 mM), and the FL intensity was measured at an excitation of 464 nm and emission of 600 nm. The second one was PBA, where 150 µL of different concentrations of PARIHN (0.00375–37.5 nM) was added to the microplate wells, followed by the addition of 150 µL of PBA (15 mM) in PBS (pH 7.4), and the FL intensity was measured at an excitation of 495 nm and emission of 590 nm.

### 2.7. ELISA vs. PARIHN-LISA for Determination of SARS-CoV-2 Nucleoprotein

A commercially available 96-well sandwich ELISA kit for the quantitative detection of SARS-CoV-2 nucleoprotein was reformed to be applied to PARIHN-LISA. Each well of the ELISA plate was pre-coated with a target-specific capture Ab in the kit, and upon the addition of standards containing SARS-CoV-2 nucleoprotein as the target antigen, they bind to the capture Ab. After washing, 100 µL of PARIHN (detection Ab), which binds to the captured Ag, was added and incubated for 1.0 h at 37 °C. Then, the plate was washed five times with washing buffer; then, 100 µL of 150 µL of 15.0 mM of PBA or 50.0 mM of boric acid was added to the microplate, respectively. Lastly, the FL intensity was measured after 1 min of the addition of PBA or boric acid by the microplate reader.

For evaluation purposes, colorimetric measurements, in which streptavidin–HRP complex and TMB substrate were added to the biotinylated sandwich immunocomplex, resulting in color development according to the standard ELISA manual, were performed. Then, sulfuric acid as a stop solution was added, and the absorbance was measured at 450 nm using the microplate reader.

### 2.8. Development of Immunochromatographic Assay Using Zinc PIHN for COVID-19 Through Modifying Commercially Available Rapid Testing Kit

A commercially available rapid testing kit for COVID-19 was modified with a PARIHN-labeled detection Ab instead of a gold nanoparticle-labeled detection Ab in the area above the sample pad and below the test and controlled lines. One micromolar of detection Ab of the COVID-19 PARIHN was used with different concentrations of antigens.

## 3. Results and Discussion

### 3.1. The Development, Characterization, and Optimization of PARIHN

**Figure 1:**
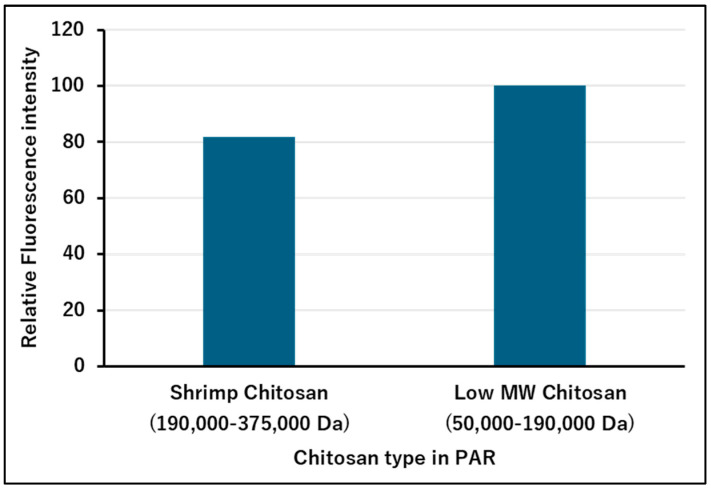
The effect of chitosan type and molecular weight on the fluorescence intensity of the reaction between PAR (0.5 μM) and boric acid.

**Scheme 2:**
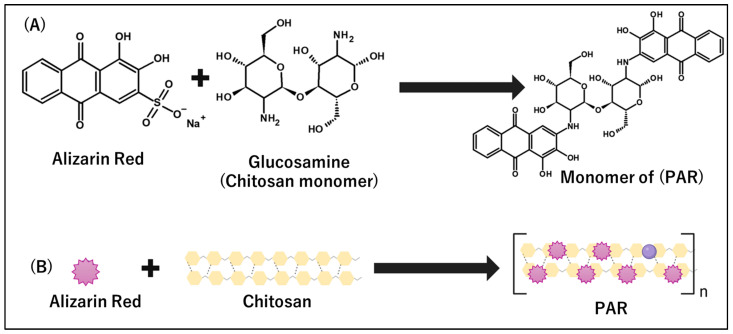
Synthesis of PAR: (**A**) simplified illustration of the nucleophilic substitution process between alizarin red and chitosan, which forms PAR by displacing the sulfonate group, and (**B**) sketch of the formation of PAR.

At first, PAR was synthesized from the reaction between alizarin red and deacetylated chitosan (Scheme 2). In the beginning, two different types of chitosan were tried, namely, low-molecular-weight chitosan (50–190 kDa) and shrimp chitosan (190–375 kDa). The low-molecular-weight chitosan was chosen to be used as it has better solubility than shrimp chitosan, and its produced polymer with alizarin gave higher fluorescence intensity with boric acid than that of shrimp chitosan–alizarin polymer (Figure 1).

**Figure 2:**
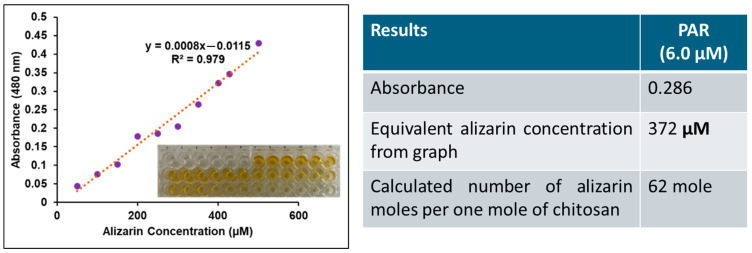
Calculation of the number of alizarin red molecules attached to chitosan by measuring the absorbance of alizarin red in the PAR after its selective reaction with PBA at 480 nm.

As described in the experimental Section, a deacetylated low-molecular-weight chitosan was reacted with alizarin red to produce PAR. The number of alizarin red moieties attached to chitosan was measured using colorimetric methods through the reaction between PBA and chitosan–alizarin through the alizarin dihydroxy group to give an orange color at 480 nm, as explained in Figure 2. There were 62 alizarin molecules attached to one chitosan molecule in the polymer.

Next, we decided to use the unique ability of polymer–IHN to directly attach a detection Ab with zinc as an inorganic core and PAR forming PARIHN to enhance the sensitivity and simplicity of immunoassay. The PARIHN was synthesized with Zn^2+^ metal, as described in the experimental Section. The selection of zinc was based on its role in the application, where Zn^2+^ was the best fit for FL signaling, which will be discussed in detail later. PARIHN was synthesized by first mixing a secondary Ab in a PBS (pH 7.4) solution that contains a metal (zinc acetate), which maintains a stable pH and provides the needed environment for the reaction. The metal ions (Zn^2+^) work to provide the necessary building blocks for the PARIHN structure with the Ab and organic polymer compound PAR. Then, the metal ions will nucleate and grow around the proteins (secondary Ab) and organic compounds (PAR), forming a nanostructure known as PARIHN. The formation of our PARIHN is similar to that reported in the literature for the formation of PIHN [17,18,19,33,34].

The detailed mechanism and role of Zn(II) in facilitating antibody conjugation to the chitosan–alizarin polymer is through coordination with the functional groups of the antibodies, such as amides and amino groups, which stabilize the antibodies. Moreover, zinc is also involved in the crosslinking mechanism between antibodies and the chitosan–alizarin red polymer, where it can bridge/link antibody [35] amide and carboxylic groups [36,37] to alizarin red catechol and quinone groups [38], and to chitosan vicinal amino and hydroxy groups [39,40]. This interaction enhanced the stability of the antibody–chitosan–alizarin red conjugate. This is in accordance with previous reports highlighting stable complexes of Zn(II) with chitosan [39,40], Zn(II) with alizarin [38], and Zn(II) with antibodies [35]. In summary, Zn^2+^ plays a critical role in the binding of antibodies to organic labels in the PARIHN through coordination chemistry, stabilization, and electrostatic interactions. These mechanisms not only enhance the structural integrity and bioactivity of hybrid materials but also improve their functionality in various biomedical applications.

**Figure 3:**
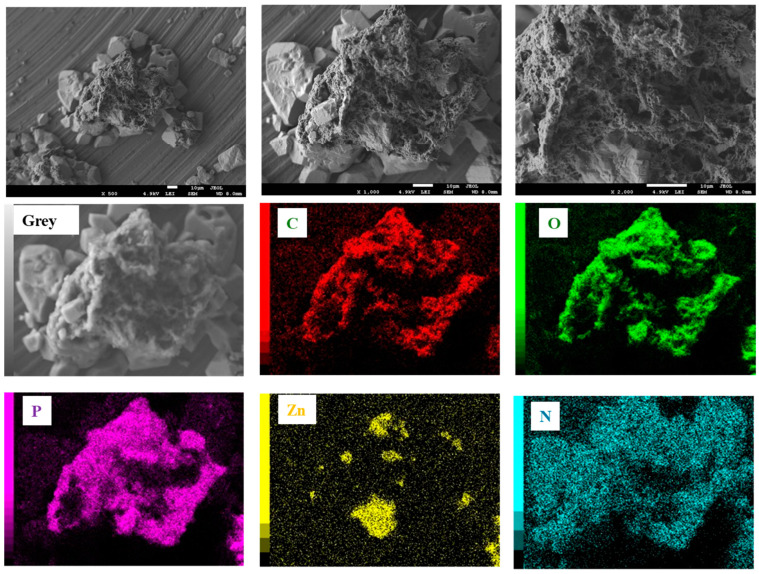
SEM images of Zn–Ab–PAR–IHN abbreviated as PARIHN with EDS-elemental mapping of C, O, P, Zn, and N at 15.0 KV.

The concentration of the organic component (PAR) and metal (Zn^2+^) was optimized, and 1 mg/mL of chitosan–alizarin and 1.0 mM Zn^2+^ were chosen. Moreover, the ratio of protein (Ab secondary) to organic components (chitosan–alizarin polymer) was determined by measuring the color intensity of alizarin at 560 nm for 1.0 μM PARIHN-Ab and calculated to be 1 Ab:16 organic components (PAR). The nanostructure shape of the PARIHN was observed by scanning electron microscopy (SEM), where in Figure 3, a nanoarchitecture structure was revealed. According to the energy dispersive spectroscopy (EDS) elemental mapping, C, Zn, N, O, and P are well distributed within the PARIHN.

**Figure 4:**
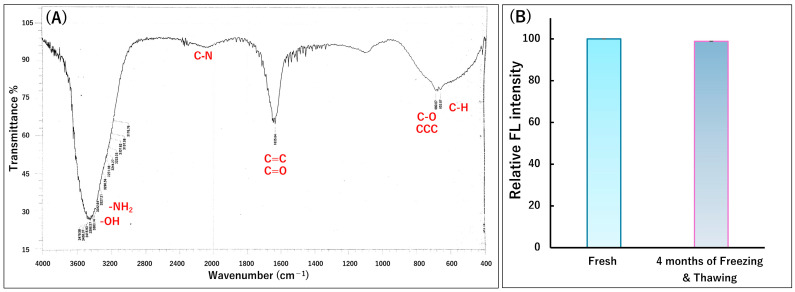
(**A**) The FT-IR spectrum of PARIHN and (**B**) the stability study of the PARIHN after four months with freezing and thawing.

Afterward, we conducted FT-IR measurements for the PARIHN, and the results are shown in Figure 4A. There are multiple peaks at 3200 to 3600 cm^−1^, which corresponds to the stretching peaks of OH and NH groups of chitosan [39,40] and the catechol groups of alizarin red [38]. The peaks at 1635 cm^−1^ and 1680 cm^−1^ correspond to C=C and C=O groups of alizarin red [38]. The peaks at 653 cm^−1^ and 680 cm^−1^ correspond to the bending peak of C-H [39] of chitosan and C-O and CCC [41] peak of alizarin red, respectively. Next, as a stability test for PARIHN in PBS buffer pH (7.4), the FL intensity was measured upon reaction with PBA after repeated freezing and thawing processes for four consecutive months, as shown in Figure 4B. The results show that PARIHN is extremely stable in PBS.

### 3.2. Study of the Reaction of PAR and PARIHN with Boric Acid Derivatives

**Scheme 3:**
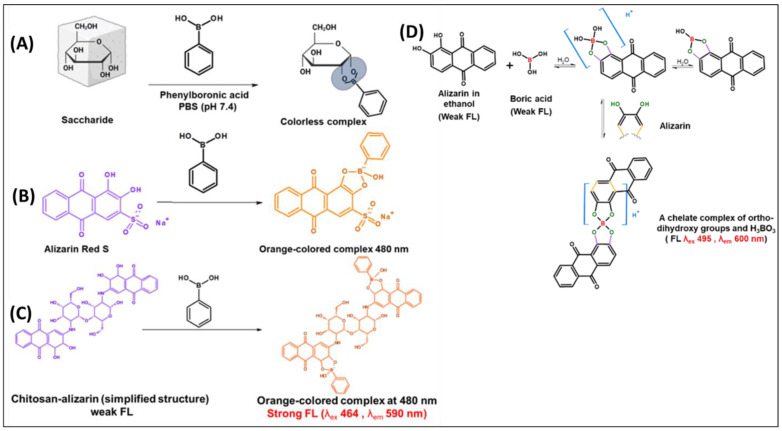
Illustrating (**A**) the reaction of PBA and any saccharide, (**B**) the reaction between PBA and alizarin, (**C**) the reaction between PBA and chitosan–alizarin polymer showcasing both colorimetric and fluorescent detection wavelength, and (**D**) the proposed mechanism of the reaction of boric acid with alizarin.

The FL assay of PARIHN is performed via the reaction of the dihydroxy groups in alizarin with PBA or boric acid. The mechanism between PBA and alizarin is well documented in several articles, where boronic acid is known to have an affinity to bind to 1,2 diol-containing compounds such as alizarin (Scheme 3A–C), where it can be used in both colorimetry at 480 nm and FL measurement at λex 464 nm and λem 590 nm [42,43,44].

**Figure 5:**
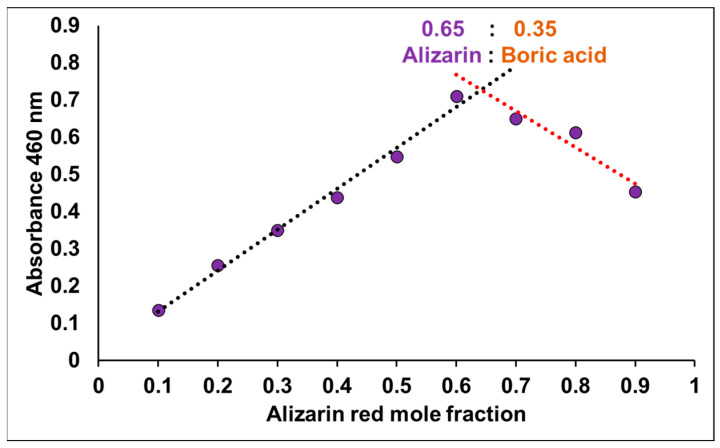
Job’s plot for the reaction between chitosan–alizarin and boric acid, where the black line is the absorbance increase trend line, and the orange line indicates the absorbance decrease trend line, both intersecting at nearly 0.65 indicating the ratio of 2:1, alizarin: boric acid.

The boron moiety in PBA in PBS (pH 7.4) is an electron-deficient “Lewis acid,” and alizarin has a partially deprotonated hydroxyl group that is electron-rich “Lewis base” and can react together to form a boronated ester complex orange in color Scheme 3B [43,44]. The formed boronated ester complex is thermodynamically favorable, and it changes the electronic structure of the alizarin molecules, resulting in FL ability [44]. PBA and other boronic acids are commonly employed due to their higher affinity for cis-diol-containing compounds. Still, the direct use of boric acid (λex 495 nm, λem 600 nm) as a reagent assay was not tried in the literature, according to our knowledge, and its mechanism is not well-known. We have proposed a mechanism in Scheme 3D based on a similar mechanism proposed by Wang et al. between carminic acid and borax-HCl buffer [45]. We employed Job’s plot method to prove the proposed mechanism for each boric acid molecule, and from the results obtained in Figure 5, it was found that two alizarin molecules might attach to one boric acid molecule. The stoichiometric ratio is found to be 0.65 alizarin:0.35 boric acid following the proposed mechanism. The overall mechanism can generally be explained as follows: when alizarin, which acts as a fluorophore, reacts with PBA/boric acid, it forms a boronated ester complex (organ-colored complex), as shown in Scheme 3 [46]. The boronated ester complex results in changes in the emission characteristics of alizarin and, thus, the shifts in the wavelengths of the emitted light and FL intensity [43,44,46,47].

Before incorporating the organic compounds (PAR) into zinc PARIHN, its reaction with boric acid and PBA was optimized, and a calibration curve was constructed using the two reagents. The results are summarized in Table 1. PARIHN showed a linear response to the fluorogenic reactions with boric acid and PBA in the range of 3.0–300.0 nm with an LOD of 0.04 and 0.10 nM, respectively, which are very promising for further application in immunoassay labeling.

**Figure 6:**
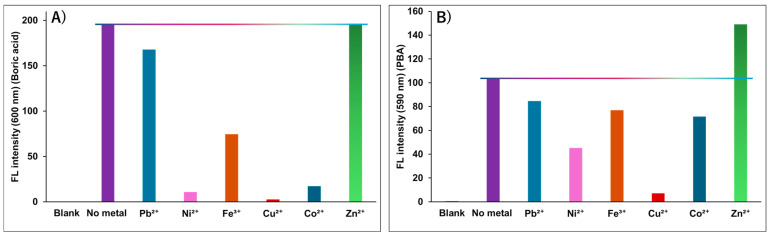
Effect of different metals on the fluorogenic reaction of PAR with (**A**) boric acid and (**B**) PBA. Where no metal, Pb^2+^, Ni^2+^, Fe^3+^, Cu^2+^, Co^2+^, and Zn^2+^ are represented by violet, blue, pink, orange, red, dark blue, and green columns, respectively.

Additionally, different metals were tested to check their influence on the fluorogenic reaction between PAR and the boric acid derivatives and to choose the best metal to use as the inorganic core for PARIHN. The tested metals included Zn^2+^, Pb^2+^, Ni^2+^, Fe^3+^, Co^2+^, and Cu^2+^. The results are summarized in Figure 6. Only Zn^2+^ resulted in no decrease in the fluorescence output of the reaction between PAR and boric acid, while Pb^2+^ led to a slight decrease in fluorescence. On the other hand, other metals decreased the fluorescence output of the reaction (Figure 6A). For the reaction between PAR and PBA, only zinc increases the fluorescence output of the reaction, while other metals lead to a fluorescence decrease with a minimum effect from Pb. From these results, it can be concluded that Zn is the best metal to be used for the synthesis of PARIHN, followed by Pb, as these two metals are diamagnetic in nature and, hence, have no or minimal quenching effect on fluorescence [48,49].

Other metals, including Ni^2+^, Fe^3+^, Co^2+^, and Cu^2+^, have a paramagnetic nature owing to the presence of unpaired electrons in their electronic configurations, which can interact with the excited states of the PARIHN boric acid derivative fluorescence complex. Additionally, the unpaired electrons in these paramagnetic metals could lead to spin-orbit coupling, which can mix singlet and triplet states. This interaction can facilitate non-radiative transitions and effectively deactivate the excited state. Additionally, their paramagnetic cations can promote intersystem crossing to triplet states [50], which are often longer-lived than the singlet states. This can result in a decrease in fluorescence as the molecule spends more time in a nonemissive state. Furthermore, these cations can also accept energy from the excited state of the organic molecule, leading to nonradiative energy transfer, which quenches fluorescence [48,50,51,52,53]. Finally, diamagnetic metals, including Zn^2+^ and Pb^2+^, can be used for the synthesis of PARIHN. However, Pb^2+^ is toxic [54] and yields lower fluorescence than Zn^2+^. Consequently, Zn^2+^ was used as an inorganic component in the formation of PARIHN.

Next, both PBA and boric acid reaction parameters with PARIHN were optimized, and it was found that maximum fluorescence intensity was obtained when PBA and boric acid concentrations were 15.0 mM and 50.0 mM, respectively. Lastly, the analytical performance of the prepared Zinc PARIHN (0.05–10.0 nM) was assessed by measuring its reaction product with PBA and boric acid fluorometrically, and the LOD with PBA and boric acid was found to be 15.9 pM and 2.6 pM, respectively, as shown in Table 1.

### 3.3. Application of Zinc PIHN in PIHN-LISA for Detection of SARS-CoV-2 Nucleoprotein

**Figure 7:**
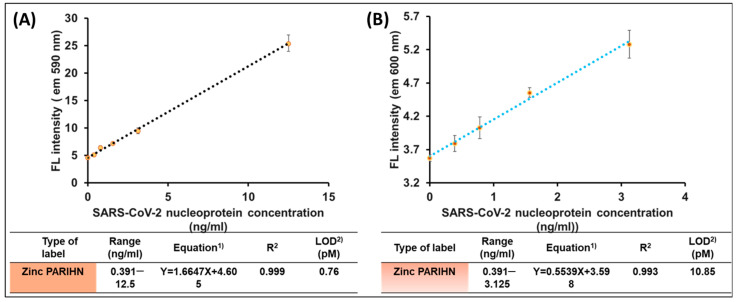
Calibration curves for (**A**) SARS-CoV-2 nucleoprotein (ng/mL) against FL intensity using Zinc PARIHN and PBA, and (**B**) SARS-CoV-2 nucleoprotein (ng/mL) against FL intensity using Zinc PARINH and boric acid. Where ^1)^ the regression equation where Y is the FL intensity and X is the PARIHN concentration in ng/mL, ^2)^ LOD = 3SD/Slope.

To illustrate the reliability of the developed label, zinc PARIHN was applied to detect the SARS-CoV-2 nucleoprotein, a basic structural protein of the COVID-19 virus [55]. The standard method for its measurement relies on a sandwich-principle-based ELISA kit for SARS-CoV-2 nucleoprotein, where the amount of SARS-CoV-2 nucleoprotein is proportional to the addition of an enzyme/chromogen (streptavidin–HRP/TMB) to the formed sandwich immunocomplex. The color of the enzymatic reaction is observed when the TMB substrate changes from colorless to blue by HRP to yellow by the addition of sulfuric acid. The yellow complex is measured at 450 nm, where its intensity is proportional to the amount of SARS-CoV-2 nucleoprotein. On the other hand, in our method, zinc PARIHN was used instead of the enzyme to target SARS-CoV-2 nucleoprotein by labeling the detection Ab with PAR as the organic components in PARIHN and then adding either PBA or boric acid to produce FL. Furthermore, PBA FL systems are more stable and straightforward than the commonly used ELISA systems (H_2_O_2_/luminol or TMB/H_2_O_2_), which are known to be dependent on ROS and easily disrupted by other factors. All other conditions were optimized and carried out in the immunoassay application. Known concentrations of SARS-CoV-2 nucleoprotein were used to prepare a calibration curve, where FL intensity for zinc PARIHN was plotted against the concentration of standards (ng/mL). A linear relationship between FL intensity and concentration was observed, as shown in Figure 7. The LOD defined at 3σ/slope, where σ is the blank standard deviation, was found to be 0.765 and 10.85 pm for PBA and boric acid, respectively, while the colorimetric ELISA method had an LOD of 7.88 pM, as shown in Figure 7.

Additionally, the zinc PARIHN sensitive reaction with PBA had a very sensitive LOD; however, the conventional ELISA was more sensitive than boric acid because it contains ethanol, which is harsh to the plastic microplate and causes the signals to be lower.

We evaluated the recovery percentage of our newly developed labels and standard ELISA for SARS-CoV-2. Our results demonstrate a high recovery percentage, as shown in Table 2. These findings indicate a high level of accuracy and efficiency of the proposed nanoflowers.

To further demonstrate the validity of the developed PARIHN-LISA methods using PBA and boric acid, they were statistically compared with the commercially available ELISA method. First, the Brown–Forsythe test and Bartlett’s test were employed to verify variance homogeneity and equality [56,57], and it was found that PARIHN-LISA and ELISA techniques had similar variances, demonstrating the proposed methods’ precision for SARS-CoV-2 NP Ag measurement (Table 2). The ANOVA test was used to compare the recovery rates of the PARIHN-LISA and ELISA techniques. It was found that there is no significant difference between our developed PARIHN-LISA method and the reference ELISA method in terms of recovery rates and accuracy.

### 3.4. Performance of Zinc PIHN in Immunochromatographic Assay

**Scheme 4:**
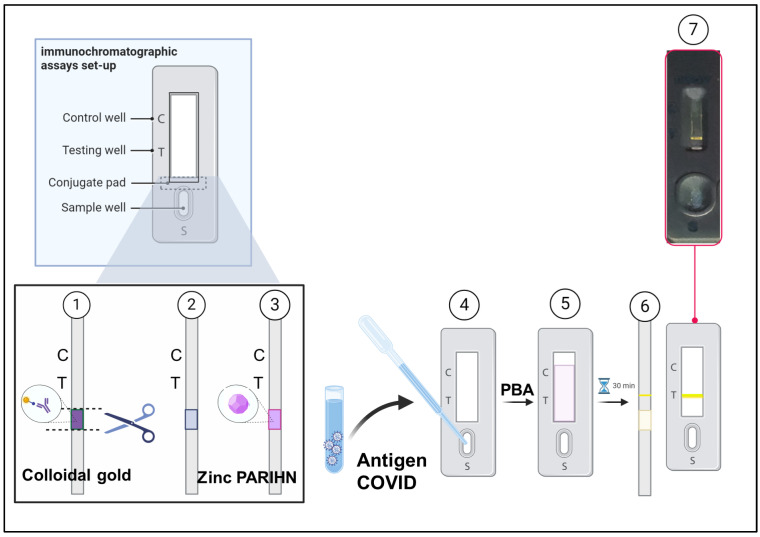
Illustration of a commercially available ICA design showcasing the traditional setup design with control and testing line, conjugated pad, and sample well. It also illustrates the general replacement of colloidal gold with Zinc PARIHN in the membrane and the subsequent fluorescent development upon adding the antigen “COVID” to the strip: (**1**) The traditional colloidal gold typically used in ICA is cut out of the membrane. (**2**) The membrane without colloidal gold. (**3**) Zinc PARIHN replaces the colloidal gold in the membrane. This modification enhances the sensitivity and detection capabilities. (**4**) The sample containing the antigen “COVID” is applied to the strip. (**5**) Followed by reagent addition after the COVID sample has migrated across the membrane, PBA is added to initiate an FL reaction, and (**6**) after 30 min, a visible bright yellow/orange fluorescent line is generated at 245 nm. (**7**) This FL line indicates the presence of the target antigen (COVID).

**Figure 8:**
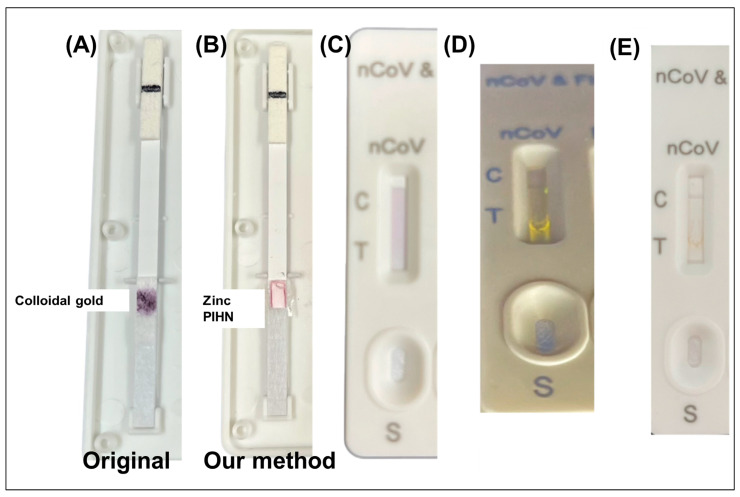
Modification of the commercially available gold test strip with Zinc PARIHN and comparison of the results with the naked eye: (**A**) Original immunochromatographic strip with colloidal gold. (**B**) Our method, where the colloidal gold was removed and replaced with Zinc PARIHN. (**C**) After adding the SARS-CoV-2 nucleoprotein-positive solution to Zinc PARIHN. (**D**) SARS-CoV-2 nucleoprotein-positive test using Zinc PARIHN at 245 nm. (**E**) The SARS-CoV-2 nucleoprotein-positive test was performed using Zinc PARIHN in normal lighting.

To further display zinc PARIHN reliability and applicability in different immunoassays, it was applied with a simple optimization to detect SARS-CoV-2 nucleoprotein in a commercially available rapid immunochromatographic assay (ICA) testing kit for COVID-19. The COVID-19 kit is an immunochromatographic test that can diagnose COVID-19 through the detection of SARS-CoV-2 nucleoprotein in 5 min using colloidal gold. Colloidal gold is usually labeled with different dyes to facilitate visual detection, or they develop color owing to their unique optical properties. The COVID-19 concentration was reflected by the intensity of the color on the test strip. In our method, the colloid gold was replaced with zinc PARIHN under ultraviolet light of 254 nm to show a yellowish fluorescent emission, as shown in Scheme 4 and Figure 8.

**Figure 9:**
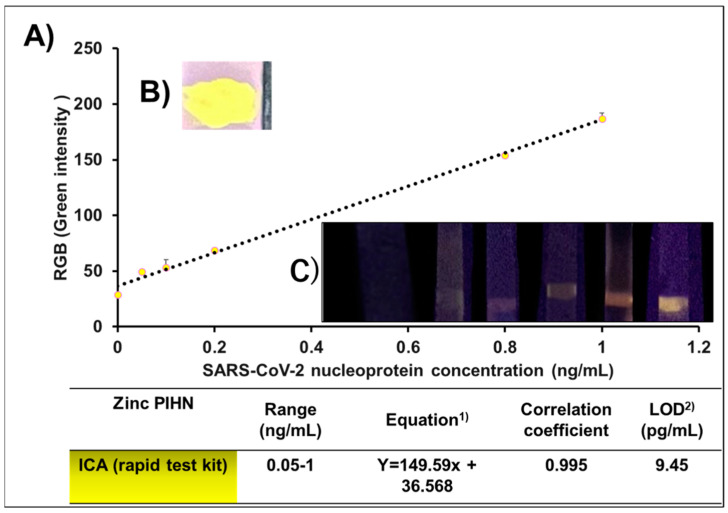
(**A**) Calibration curve SARS-CoV-2 nucleoprotein (ng/mL) against RGB (green intensity) using Zinc PARIHN and PBA. (**B**) A picture of the vivid yellow FL of 1 ng/mL of Zinc PARIHN and PBA under 254 nm, and (**C**) Quantitative FL assay of SARS-CoV2 N protein using the developed test strip under UV lamp, where from right to left concentration of COVID-19 are 0, 0.05, 0.1, 0.2, 0.8, and 1.0 ng/mL. Where ^1)^ the regression equation where Y is the FL intensity and X is the PARIHN concentration in ng/mL, ^2)^ LOD = 3SD/Slope.

**Figure 10:**
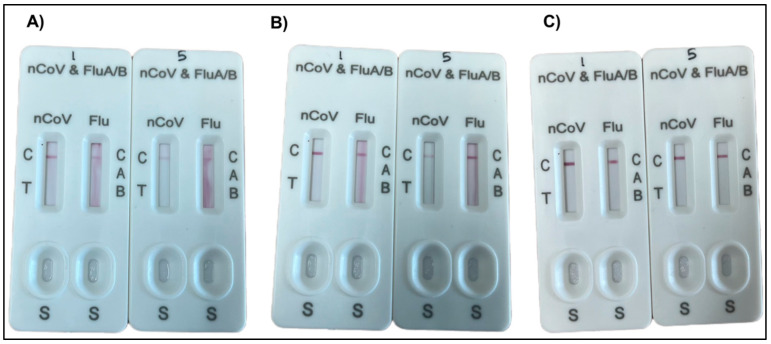
The original kit when using 1 and 5 ng/mL of SARS-CoV-2 nucleoprotein: (**A**) One minute of adding one ng/mL solution to the left and five ng/mL to the right, (**B**) after 5 min, and (**C**) after drying completely.

**Figure 11:**
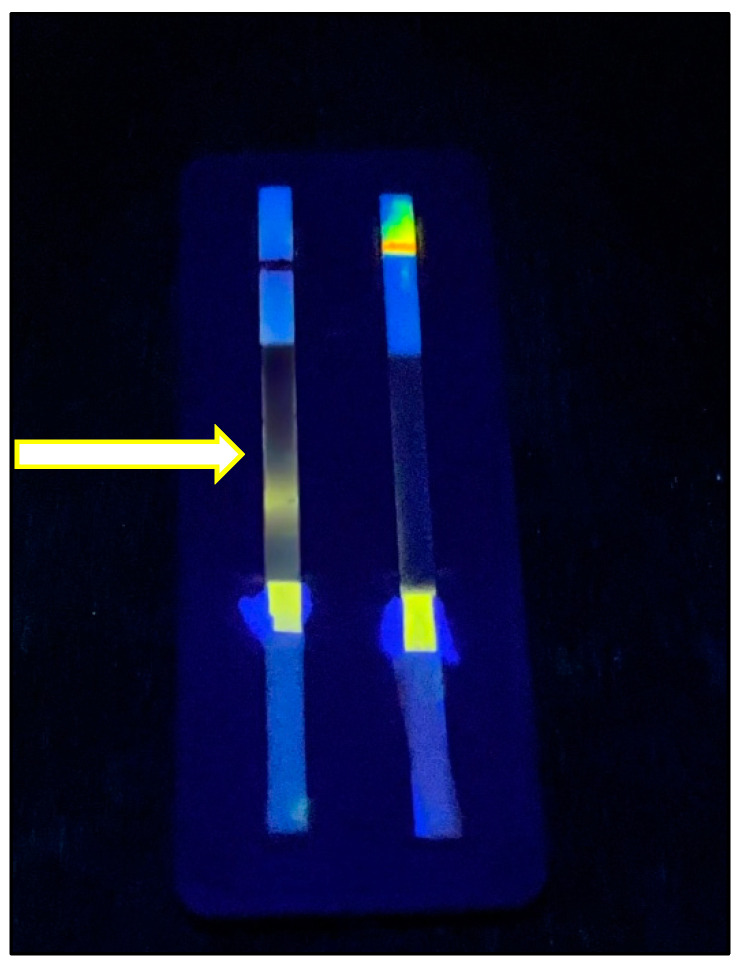
Selectivity of the developed test strip, where on the left side, the fixed capture Ab is for SARS-CoV-2 while on the right side, two Abs for influenza A and B were fixed. As can be seen, the FL signal only develops on the left side (indicated by the arrow).

A pilot quantitative study from the range of 0.05–1 ng/mL SarsSARS-CoV-2 protein was carried out using PARIHN, and the results are summarized in Figure 9. The calibration curve was obtained by plotting a graph between the green intensity of RGB against the concentration of SARS-CoV-2 protein, and excellent sensitivity was obtained down to an LOD of 9.45 pg/mL. It is worth mentioning that the original kit could not detect one ng/mL of SARS-CoV-2, as shown in Figure 10. Next, the recoveries of five samples spiked with SARS-CoV-2 were evaluated and summarized in Table 3. The recovery percentages ranged from 97.1 to 104.62%, and the RSD% ranged from 0.84 to 12.92, demonstrating the high accuracy and acceptable precision of the developed fluorescence PARIHN-based immunochromatographic assay. Furthermore, we investigated the response to influenza A/B and found that our Zn^2+^ PARIHN is selective toward SARS-CoV-2, as shown in Figure 11.

### 3.5. Challenges and Future Directions of PARIHN as Labeling Tag for Immunoassays

PARIHN technology presents an innovative and promising non-enzymatic approach for sensitive COVID-19 detection, offering potential advantages over traditional methods in terms of sensitivity, stability, versatility, cost, and speed. The high sensitivity demonstrated in both the standard immunoassay and ICA formats is a significant achievement. However, for this technology to transition from the research lab to practical clinical applications, further research must address challenges related to manufacturing scalability, rigorous clinical validation in diverse patient samples, long-term stability, real-world cost-effectiveness, and regulatory requirements. Future work exploring its adaptability for detecting other pathogens and biomarkers will be the key to realizing its full potential in diagnostic technologies. Conducting real-world trials to evaluate performance across diverse populations is essential. Additionally, combining the PARIHN with microfluidics or point-of-care devices for rapid testing will help in the development of diagnostics. Finally, transitioning from a promising research finding to a commercially available diagnostic test involves rigorous validation according to regulatory standards (e.g., FDA and European conformity marking), which is a significant undertaking.

## 4. Conclusions

In this study, we successfully developed a novel non-enzymatic fluorescent labeling system using a polymerized alizarin red–inorganic hybrid nanoarchitecture (PARIHN) for the efficient detection of SARS-CoV-2. The integration of this advanced nanoarchitecture into immunoassays and immunochromatographic assays significantly enhances sensitivity, reduces costs, and promotes environmental sustainability. Our findings demonstrate the stability and efficacy of PARIHN, achieving remarkable limits of detection for phenylboronic acid and boric acid, as well as for SARS-CoV-2 nucleoprotein. This innovative approach not only addresses the limitations associated with traditional enzymatic methods but also provides a practical solution for rapid and reliable COVID-19 diagnostics. The promising performance of PARIHN underscores its potential for broader applications in immunoassays, paving the way for future advancements in diagnostic technologies. Further research may explore the expansion of this methodology to other pathogens, contributing to enhanced public health responses.

## Figures and Tables

**Table 1:** PAR and Zn^2+^ PARIHN with their respective range, LOD, and reagents in colorimetric and fluorescence assays.

Compound	Range	Correlation Coefficient	LOD (nM)	Detection Method	Used Reagent
PAR	150.0–30,000	0.996	27.6	Colorimetric	PBA
PAR	3.0–300.0	0.998	0.10	FL	PBA
PAR	3.0–300.0	0.999	0.04	FL	Boric acid
Zn^2+^ PARIHN	0.05–10.0	0.977	0.016	FL	PBA
Zn^2+^ PARIHN	0.05–10.0	0.987	0.003	FL	Boric acid

**Table 2:** Comprehensive overview of used labels in detecting SARS-CoV-2 nucleoprotein, along with their LOD (ng/mL and pM), concentrations (ng/mL), and recovery %.

Type of Label Used	Type of Assay and Reagent	LOD (ng/mL)	LOD (pM)	Concentrations (ng/mL)	Recovery % ± SD
PARIHN	FL and PBA	0.037	0.76	0.391	97.3 ± 4.8
				0.783	108.0 ± 5.8
				1.563	99.5 ± 3.2
				3.125	96.8 ± 5.6
				12.5	100.2 ± 5.9
PARIHN	FL and boric acid	0.521	10.85	0.391	100.6 ± 3.1
				0.783	100.0 ± 4.1
				1.563	97.9 ± 1.6
				3.125	100.8 ± 3.9
Streptavidin–HRP complex	Colorimetric and TMB/H_2_O_2_	0.378	7.88	0.391	103.0 ± 7.0
				0.783	98.5 ± 4.2
				1.563	95.9 ± 4.3
				3.125	101.5 ± 1.7
				12.5	99.30 ± 0.9
Brown–Forsythe test (*p*)					0.4777 *
Bartlett’s test (*p*)					0.1578 *
One-way ANOVA (*p*)					0.9372 *

* No significant difference as *p* > 0.05.

**Table 3:** Results of recovery study of SARS-CoV2 N protein from spiked samples using the developed fluorescence immunochromatographic assay.

Tested Concentration (ng/mL)	Recovery %	RSD
0.05	104.63	1.82
0.1	97.13	12.92
0.2	99.59	2.76
0.8	98.58	0.84
1.0	101.01	2.67

## Data Availability

Most of the data are shown in the original manuscript, and if further data are needed, they will be available upon reasonable request.

## Abbreviations

The following abbreviations are used in this manuscript:

PARIHN	Polymerized Alizarin red–inorganic hybrid nanoarchitecture
PAR	Polymerized Alizarin red
ICAs	immunochromatographic assay
QCCC	quinone–chitosan conjugation chemistry
PBA	phenylboronic acid
ROS	reactive oxygen species
FIAs	Fluorescence immunosorbent assays
PIHNs	protein–inorganic hybrid nanoarchitecture
DTT	dithiothreitol
ICAs	immunochromatographic assays
HRP	horseradish peroxidase
PBS	Phosphate-buffered saline
EDS	energy dispersive spectroscopy
